# Time-lapse imaging of HeLa spheroids in soft agar culture provides virtual inner proliferative activity

**DOI:** 10.1371/journal.pone.0231774

**Published:** 2020-04-17

**Authors:** Reiko Minamikawa-Tachino, Kiyoshi Ogura, Ayane Ito, Katsuya Nagayama

**Affiliations:** 1 Translational Medical Research Center, Tokyo Metropolitan Institute of Medical Science, Setagaya, Tokyo, Japan; 2 Department of Interdisciplinary Informatics, Graduate School of Computer Science and Systems Engineering, Kyushu Institute of Technology, Iizuka, Fukuoka, Japan; 3 Department of Mechanical Information Science and Technology, Faculty of Computer Science and Systems Engineering, Kyushu Institute of Technology, Iizuka, Fukuoka, Japan; University of Bergen, NORWAY

## Abstract

Cancer is a complex disease caused by multiple types of interactions. To simplify and normalize the assessment of drug effects, spheroid microenvironments have been utilized. Research models that involve agent measurement with the examination of clonogenic survival by monitoring culture process with image analysis have been developed for spheroid-based screening. Meanwhile, computer simulations using various models have enabled better predictions for phenomena in cancer. However, user-based parameters that are specific to a researcher’s own experimental conditions must be inputted. In order to bridge the gap between experimental and simulated conditions, we have developed an *in silico* analysis method with virtual three-dimensional embodiment computed using the researcher’s own samples. The present work focused on HeLa spheroid growth in soft agar culture, with spheroids being modeled *in silico* based on time-lapse images capturing spheroid growth. The spheroids *in silico* were optimized by adjusting the growth curves to those obtained from time-lapse images of spheroids and were then assigned virtual inner proliferative activity by using generations assigned to each cellular particle. The ratio and distribution of the virtual inner proliferative activities were confirmed to be similar to the proliferation zone ratio and histochemical profiles of HeLa spheroids, which were also consistent with those identified in an earlier study. We validated that time-lapse images of HeLa spheroids provided virtual inner proliferative activity for spheroids *in vitro*. The present work has achieved the first step toward an *in silico* analysis method using computational simulation based on a researcher’s own samples, helping to bridge the gap between experiment and simulation.

## Introduction

Cancer research models for screening have included the creation of spheroid microenvironments to test drug effects [1–3]. In one of the earlier studies using a spheroid-based screen, Friedrich *et al*. [4] pointed out that using spheroid integrity and volume monitoring through phase-contrast imaging improved agent measurement when accompanied by the security of clonogenic survival. Various researchers have continued under this concept and expanded it by changing spheroid culture systems, drugs tested, and microscopes used [5–7]. One of the studies showed that spheroid cells were viable, with altered patterns of metabolism owing to lactate dehydrogenase activity and glucose utilization [8].

Moreover, image-based monitoring of cell aggregation and microspheroid growth allows for the study of morphological changes during spheroid growth. Such techniques have the potential to provide an important source of information regarding not only the self-assembly of spheroids but also spheroid use for a wide range of biofabrication methodologies in tissue engineering. The methodology used to monitor the aggregation of individual spheroids was proposed though image analysis of microscopy data [9, 10].

Cancer is a complex disease caused by multiple types of interactions across diverse physical, temporal, and biological scales. Numerous studies on cancer have extended the context of molecular, cellular, and physiological phenomena [11]. For the integration of individual studies, computational models lend themselves to understanding and predicting complicated phenomena. These models often involve three aspects. One relates to statistical cancer models at the genomic, transcriptomic, and biomolecular pathway levels. The second aspect often involves statistically inferred network models that include mechanistic bases for signaling (including metabolism), and the third is a continuum and agent-based model of cancer cell populations and tumor progression at longer timescales [12]. Computational models of metabolism, growth, proliferation, and death of single cells are described quantitatively using equations that describe biochemical and mechanical cell–cell and cell–environment interactions and their interrelations [13]. In particular, growth and organization processes are demonstrated using diverse cell-based simulations [14]. In addition, the interactions among many cells have been simulated mechanically and biochemically in relation to their microenvironment in multicellular simulations [15, 16]. These studies have been performed using statistically determined parameters based on bioassays. However, the simulations are several steps removed from a researcher’s own experiments, as they are performed only on formulated objects. Even if the objects can be adjusted based on parameters, barriers to adjusting the parameters to precisely match specific experimental conditions will exist.

We aimed to bridge this gap between spheroid data *in vitro* and *in silico* and to investigate apparently living spheroids including virtual inner activity. For this purpose, we have developed an *in silico* analysis method with virtual three-dimensional (3D) embodiment computed using a researcher’s own samples. In the present work focusing on individual HeLa spheroid growth in soft agar culture, spheroids were analyzed *in silico* by matching growth conditions with those observed in microscopy time-lapse images. The agarose format was selected because it is a scalable technique that provides uniformly sized spheroids [10] and allows for real-time monitoring of the cell aggregation process [17]. Preceding studies [18, 19] illustrated that spheroids presented with the composition of a central necrotic core region surrounded by a zone of quiescent viable cells, followed by an outer layer of actively proliferating cells. In other words, spheroids exhibited a gradient descent toward the center for nutrients, oxygen, and metabolites, which led to the observed composition. *In silico* analysis performed 3D computational replication of spheroids whose growth curves were adjusted to those obtained from time-lapse images of spheroid growth to optimize these factors. Furthermore, the *in silico* analysis assigned each cellular particle virtual inner proliferative activity, which corresponded to whether it was a proliferating cell *in vitro*. We then validated that time-lapse images of HeLa spheroids provided virtual inner proliferative activity that replicated the composition in spheroids *in vitro*.

In the present work, we achieved the first step toward an *in silico* analysis method using 3D computational simulation based on a researcher’s own samples. This research provides a foundation to develop drug testing affording sensitivity with regard to both the appearance and virtual inner activity of living spheroids along the time course from drug addition. Moreover, these highly sensitive readouts are complementary to conventional agent measurements obtained following testing, thereby permitting the extraction of more detailed information from the drug test.

## Materials and methods

**Fig 1:**
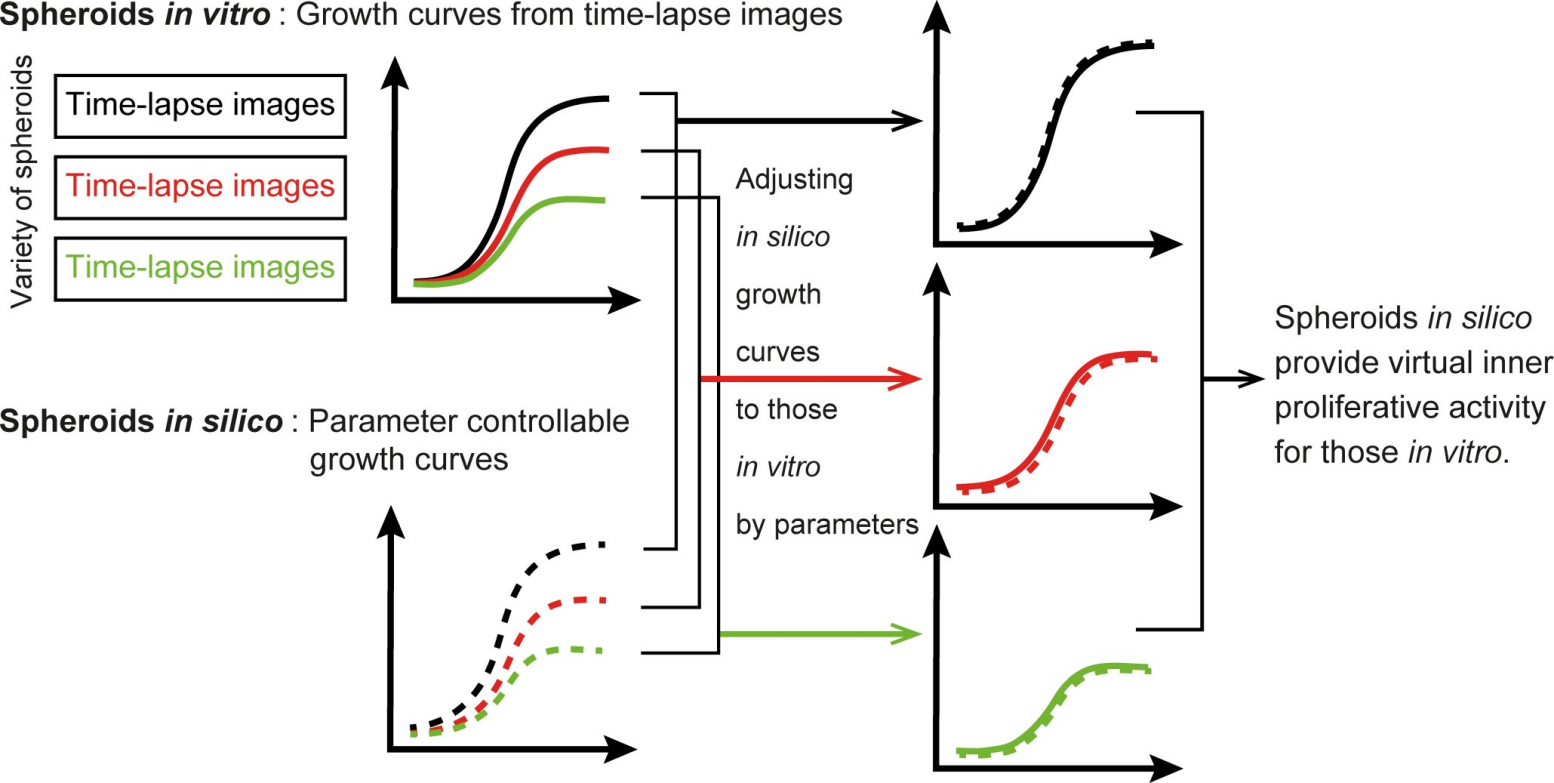
Framework to bridge between spheroids *in vitro* and those *in silico*.

The work was designed as a framework for bridging the gap between spheroid data *in vitro* and *in silico* using simulations based on experimental data (Fig 1). Virtual inner proliferative activity was examined when growth curves of spheroids *in silico* were in accordance with those of the spheroids *in vitro*.

### *In vitro* spheroid analysis

**Fig 2:**
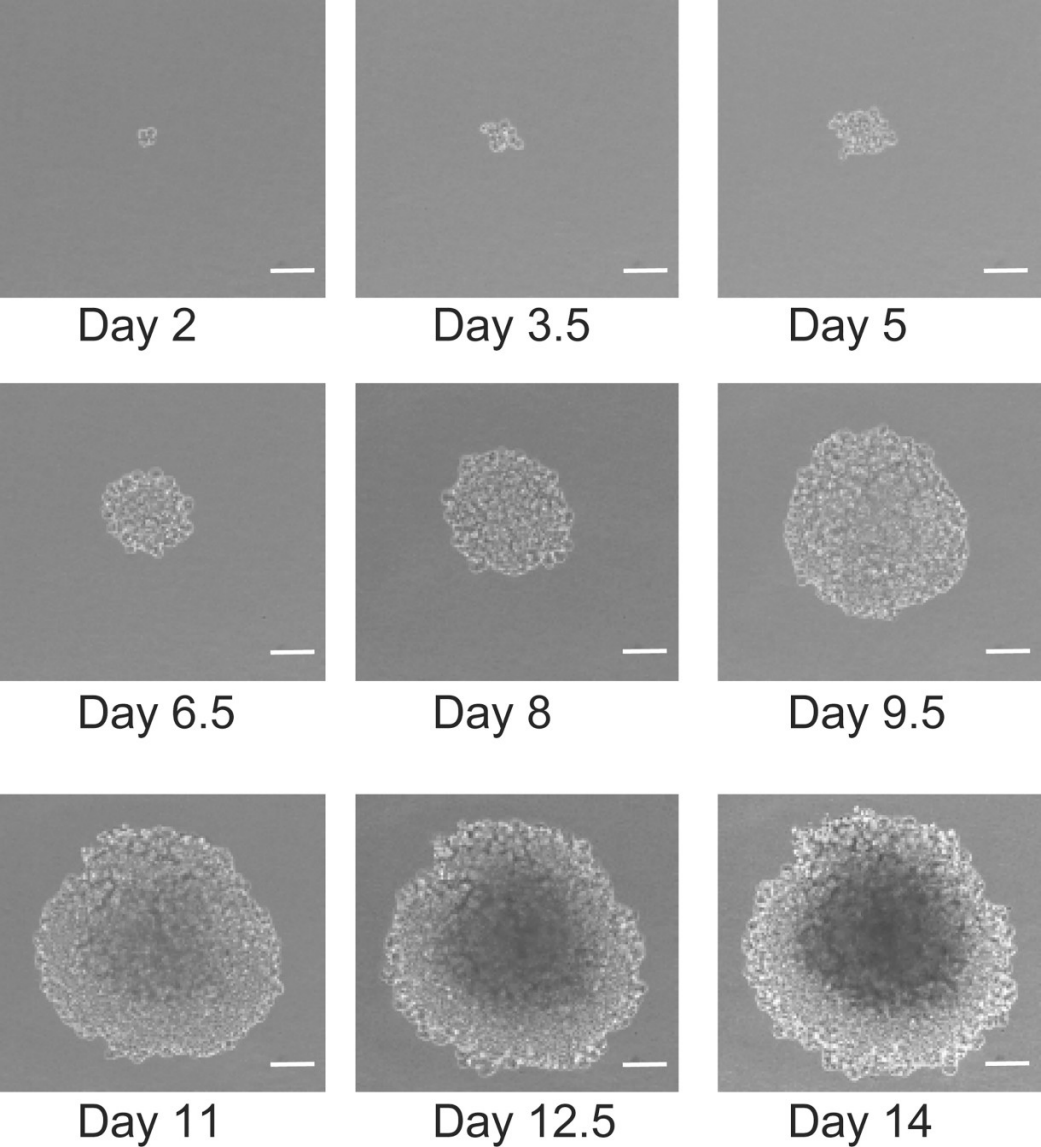
Time-lapse microscopy images of the growing process of a spheroid. The growing process was captured for each spheroid as time-lapse images by positioning the place that a cell was recognized at the center of the frame in the one-cell stage. A series of time-lapse images was chosen for the same spheroid *in vitro*, such that the spheroid was in focus over time, and its entire body was completely visible in the image and appeared alone without contacting with its surroundings over time. The scale bar is 100 μm.

The human cervical cancer cell line HeLa was obtained from Dr. Masao Kawakita at The Tokyo Metropolitan Institute of Medical Science (Rinshoken) (Tokyo, Japan) [20] on October 7, 2009. The cells were grown in soft agar in accordance with an assay protocol, a detailed description of which is shown in S1 Table. HeLa cells in 0.35% agarose medium were seeded on a solid layer of 0.7% agarose medium in a 6-well culture plate and incubated for 1 day. Seeding density was kept sufficiently low (500 cells/well) to prevent spheroids from touching each other to analyze the individual growth process of each spheroid via time-lapse imaging of its growth. After 5 mL/well of the growth medium was added, the cells were incubated for 14 days from day 2–15 in a BioStation CT (Nikon, Tokyo, Japan) composed of a transport unit for plate transportation within the incubation area and an observation unit for automated imaging. During this period, the transport unit individually conveys the plates from the storage rack to the observation stage in accordance with configured schedules. Spheroid images were captured automatically in each well at 3 h intervals with a phase-contrast microscope at 10× magnification at each time point. Fig 2 shows an example of time-lapse spheroid growth.

### Growth curves of spheroids *in vitro*

A spheroid appeared over time in each captured image of 1000 × 1000 pixels with 256 gray levels. The spheroid was segmented using ImageJ by a conventional step consisting of binarization and morphological processing. Thresholding by pixel value was used to convert each image to binary, one indicating whether the pixel belonged to the spheroid. The morphological processing was used to adjust the binary image to cover the entire spheroid and remove background noise. As a result, the binary image showed one single region in which pixels all belonged to the spheroid. Subsequently, the region was replaced as a conceptual circle with the same area as the segmented region. The present work assumed that a spheroid was a sphere based on the fact that spheroids frequently appear as spheres in scanning electron microscopy [21]. The size of a single cell was determined in the same manner from the region segmented into images of the one- or two-cell stage.

Using images, Martin *et al*. demonstrated that an aggregate consisted of cells for which the count was in the range between the lower and upper bounds calculated by 3D simple cubic lattice packing and face-centered cubic lattice packing, respectively [22]. A spheroid was assumed to consist of cellular spheres packed with a face-centered cubic lattice inside. The number of cells was estimated as the number of the single cells packed with face-centered cubic lattice structure in the spheroid sphere approximated by the replaced circles. Consequently, a growth curve of spheroids *in vitro* could be obtained by plotting this estimated number of cells.

### *In silico* spheroid analysis

The *in silico* spheroid analysis involved a spheroid growth simulation based on the lattice-free particle model, which is a numerical simulation method to evaluate the physical quantities of a particle at a calculation point. A spheroid *in silico* consisted of cellular particles that moved without lattice restriction while maintaining a tiny predefined distance, which was relative to the cell diameter. The size of an individual cellular particle was set as 10 μm, using the average size of a typical human erythrocyte (7.2 μm) as a guide [23]. This is reasonable for a relative spatiotemporal transformation between *in vitro* and *in silico* information because cells generally have bodies that are 10–30 μm in size [24].

The simulated growth sources, which consisted of nutrients and oxygen, were delivered to the outer cellular particles directly, diffused toward the center, and then supplied to cellular particles inside. A cellular particle divided in accordance with its cell cycle was controlled by the supplied growth sources and its environment.

### Models for spheroids *in silico*

A spheroid *in silico* consisted of cellular particles with heterogeneous structure comprising simple cubic structure and hexagonal close-packed structure. This structure was sustained by attractive and repulsive forces between two cellular particles. The force between the particles is determined as follows:

$$F=k\left(1-\frac{r}{R\cdot r_{0}}\right)\left(1-\frac{r}{G\cdot r_{0}}\right),$$ (1)

where *k* is a constant, *R* is a repulsion constant, *G* is an attraction constant, *r* is the distance between the particles, and *r*_0_ is the base distance. The constants were derived from the packing density in a hexagonal close-packed structure and provided balance for attractive and repulsive forces.

**Fig 3:**
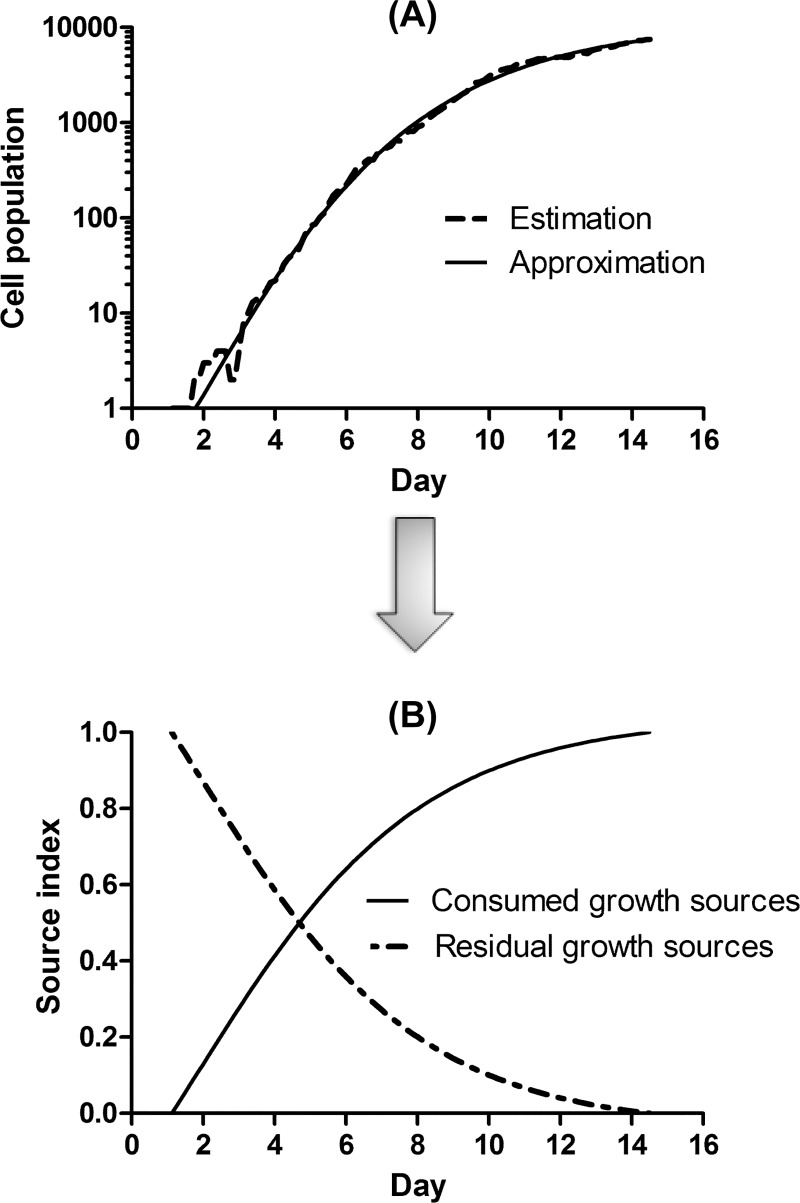
Residual growth source estimated from the growth curve. **(A)** A growth curve was estimated from the time-lapse spheroid images, drawn using a dashed line. **(B)** Growth sources were consumed corresponding to the approximated growth curve. The residual growth sources at the local level are the reverse of the consumed sources because no replenishment was carried out during incubation.

Cell population growth *in vitro* depends, in a general sense, on the growth sources that are available in the culture medium. Focusing on individual spheroid growth, the growth curves of spheroids *in vitro* allowed us to estimate decreases in growth sources locally (i.e. locally around the spheroids) during incubation. Fig 3 shows the relationship between cell population growth (Fig 3A) and changes in the growth source at the local level (Fig 3B). The residual growth source decrease depends on increases in the cell population. The residual growth sources at the local level were estimated as follows: let *C*_*t*_ be the consumed growth sources, namely, the sum of the growth sources supplied to each cellular particle at time *t*. An index *R*_*t*_ of residual growth sources is calculated as follows:

$$R_{t}=1-\frac{\sum_{k=0}^{t-1}C_{k}}{Q_{0}},$$ (2)

where *Q*_*0*_ is the initial amount of growth sources. After growth sources were supplied to the outer cellular particles, the residual growth sources diffused into the center of the spheroid. The residual growth sources were conveyed to cellular particles inside. However, it is impossible to determine the exact amount of the growth sources supplied and consumed in each cellular particle. Assuming that the consumption was fixed, the residual growth sources diffused to a cellular particle according to the following formula:

$$\frac{\partial R_{t}}{\partial t}=D\left(\frac{\partial^{2}R_{t}}{\partial r^{2}}\right)-\alpha ,$$ (3)

where *r* is the distance between the particles, *D* is the diffusion coefficient, and *α* is the particle consumption. This was repeated for inner cellular particles up to 200 μm from the outer surface of the spheroid because it has been reported that oxygen is conveyed up to approximately this distance from blood vessels [25].

A basic outline of the cell cycle was determined using the Monod equation, which relates population growth rate to nutrients [26]. In addition, the cellular environment of the particles was introduced as a mechanism of cell cycle control. The environment was defined as the density of a cellular particle, which was the number of cellular particles in its surroundings. The maximum density corresponds to the kissing number in geometry problems [27], specifically 12 in three dimensions, which is the maximal number of cells that each cell can touch without overlapping in the hexagonal close-packed structure. The density decreases toward the spheroid surface. Mobility was also introduced as a ratio of distance between the particle and the spheroid center to the spheroid radius. The mobility increases toward the surface.

The cell cycle *T*_*z*_ for a cellular particle *z* is determined as follows:

$$T_{Z}=\frac{1}{\mu }=\frac{1}{\mu_{\max}\left(1+K_{c}{}^{2}\right)}\left(1+{\left(\frac{K_{c}}{N\left(Z\right)}\right)}^{2}\right)\left(1+D_{Z}\frac{K_{e}}{P_{Z}}\right),$$ (4)

where *μ*_max_ is the specific maximum growth rate, *K*_***c***_ is a relaxation coefficient for growth sources, *N*(*z*) is the amount of growth sources conveyed to *z*, *D*_***z***_ is the density, *K*_*e*_ is a relaxation coefficient for the environment, and *P*_*z*_ is the mobility. The specific maximum growth rate was calculated by the minimum cell cycle time measured in the images in the first stage. The cell cycle was practically controlled by the two relaxation coefficients.

### Simulation for spheroid growth

Simulation was performed using a program running on a PC (CPU: Intel Core^TM^ i7 3.33 GHz; RAM: 24.0 GB). The program was developed in FORTRAN by the authors. The simulation started on day 4 to avoid an initial fluctuation in the growth curve as shown in Fig 3A. Table 1 summarizes the parameter values predefined in the execution. Growth source transport, cell division, and cell movement were simulated in 1 min steps. If a cellular particle divided, the original cell was replaced by its daughter particles. Nevertheless, the spheroid maintained a mostly hexagonal close-packed structure because cellular particles surrounding the daughter particles moved so as to keep the forces well balanced. A generation number was assigned to each daughter particle, which signified how many cell divisions preceded their production.

**Table 1:** Parameters for the simulation.

Parameter		Value
Time interval		1 min
Total time		User settings
Cellular particle diameter		10 μm
Forces between cellular particles	Constant	k = 0.005
	Repulsion constant	R = 1.106
	Attraction constant	G = 1.5
Growth sources diffusion	Maximum diffusion distance	200 μm
	Diffusion coefficient with fixed particle consumption	α/D = 20
Minimum cell cycle time		User settings

Only two parameters, total time and minimum cell cycle time, were specified by users. Parameters for forces were fixed theoretically based on the hexagonal close-packed structure. With regard to the diffusion of the growth sources, the parameters were fixed based on preliminary examinations assuming that no growth sources were beyond 200 μm away. The total time was set as 20 days and the minimum cell cycle time was 18 h in the present work.

### Bridge between spheroids *in vitro* and *in silico*

A bridge was used to adjust the *in silico* growth curves to those *in vitro* toward integration of the experiment and the simulation. This is equivalent to determining the relaxation coefficients in the cell cycle calculation. To do this systematically, an error function was introduced as follows:

$$f\left(K_{c},K_{e}\right)={\sum_{t}\left(\frac{Log\;e(t)-Log\;n(t)}{Log\;e(t)}\right)}^{2},$$ (5)

where *e*(*t*) is the number of cells estimated from an image at time *t*, and *n*(*t*) is the number of cellular particles in the spheroid *in silico* at time *t* for relaxation coefficients *K*_*c*_ and *K*_*e*_. Optimizing the relaxation coefficients corresponded to finding values for *K*_*c*_ and *K*_*e*_ that locally minimized the error on a plane with the *K*_*c*_ and *K*_*e*_ axes.

Spheroids *in vitro* varied in size and shape despite being incubated under the same conditions. The bridge provided a feature space of spheroids for visualization of the variations. Assuming that the growth curve of a spheroid *in vitro* was related to this variation, the parameters used in approximating the growth curves as a mathematical function could be used to characterize the spheroids according to a solid statistical foundation with principal component analysis (PCA) in machine learning [28].

A growth curve was approximated using the generalized logistic curve with five parameters, known as a Richard’s curve [29] as follows:

$$y=A+\frac{C}{{\left(1+Te^{‐B(x‐M)}\right)}^{\frac{1}{T}}},$$ (6)

where *A* is the lower asymptote, *B* is the growth rate, *C* is the upper asymptote, *M* is the time of maximum growth, and *T* is a parameter that fixes the inflection point. Parameters *B* and *M* are involved in the exponential phase in sigmoid graphs of the population growth curve, and especially with the transitional phase toward the plateau phase.

Optimization by nonlinear least squares (Solver tool, Excel^®^; Microsoft Corp., Redmond, WA, USA) provided a five-dimensional vector associated with the growth curve. As for each parameter, centering and scaling were applied to value sets of spheroids *in vitro* to normalize the value distribution. Characterization of the growth curves was performed by PCA, using a program written in C by the authors.

## Results

### Growth features in spheroids *in vitro*

**Fig 4:**
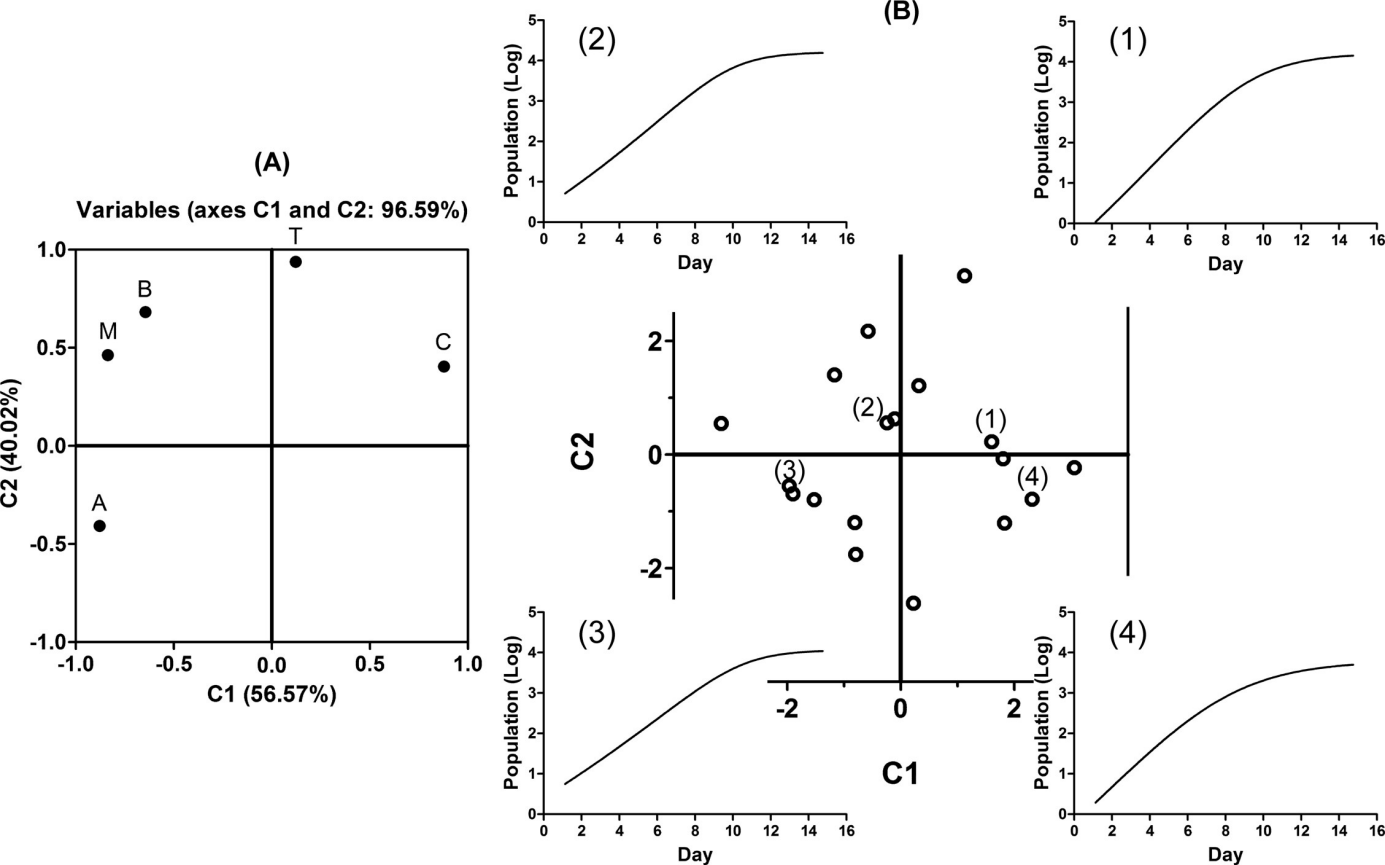
Approximated growth curves plotted on the feature plane. **(A)** Principal components were selected in order of largest contribution to the eigenvalues. The contribution rate was 56.57% for the first principal component (C1) and 40.02% for the second (C2), with the accumulated rate of 96.59%. The eigenvectors corresponding to these components formed a feature plane as the eigenspace. The factor-loading plot demonstrates that Richard’s curve parameters correlate C and T, B and M, and A with the first, second, and third quadrants of the plane, respectively. The fourth represents no correlation with the parameters. **(B)** Each approximated growth curve is plotted as a point on the feature plane by C1 and C2. The origin of the plane is the average of the growth curves because of normalization with centering among each parameter. For points (1)–(4), the Richards’ curves generated by parameters of the points are illustrated with the same number.

By applying PCA to five-dimensional vectors associated with growth curves obtained from 18 spheroids *in vitro*, a feature plane was defined as the eigenspace. These spheroids were chosen from multiple wells in different plates, because they were in focus in the series of time-lapse images, and each entire body was completely visible and alone, without contact with its surroundings, during the time of analysis. In Fig 4A, the factor-loading plot shows correlations between parameters of the Richards’ curve and each quadrant of the feature plane. Fig 4B shows the variation in growth on the feature plane and the relationships between the quadrants and the graphs of the Richard’s curves using (1)–(4). Point (2) is representative of the second quadrant and is the closest to the origin. Points (1), (3), and (4) are typical points that are surrounded by many other points in the first, third, and fourth quadrants, respectively. The transitional phase was clearest at (2) on the second quadrant, followed by (1) on the first, (3) on the third, and then (4) on the fourth. The feature plane lends itself to the identification of variation in the spheroids.

### Growth features in corresponding spheroids *in silico*

The bridge took approximately 5 h 20 min to optimize a spheroid *in silico* on day 15, with relaxation coefficients taken into account in the cell cycle calculation. The optimization was performed by changing values approximately 15 times for each spheroid.

**Fig 5:**
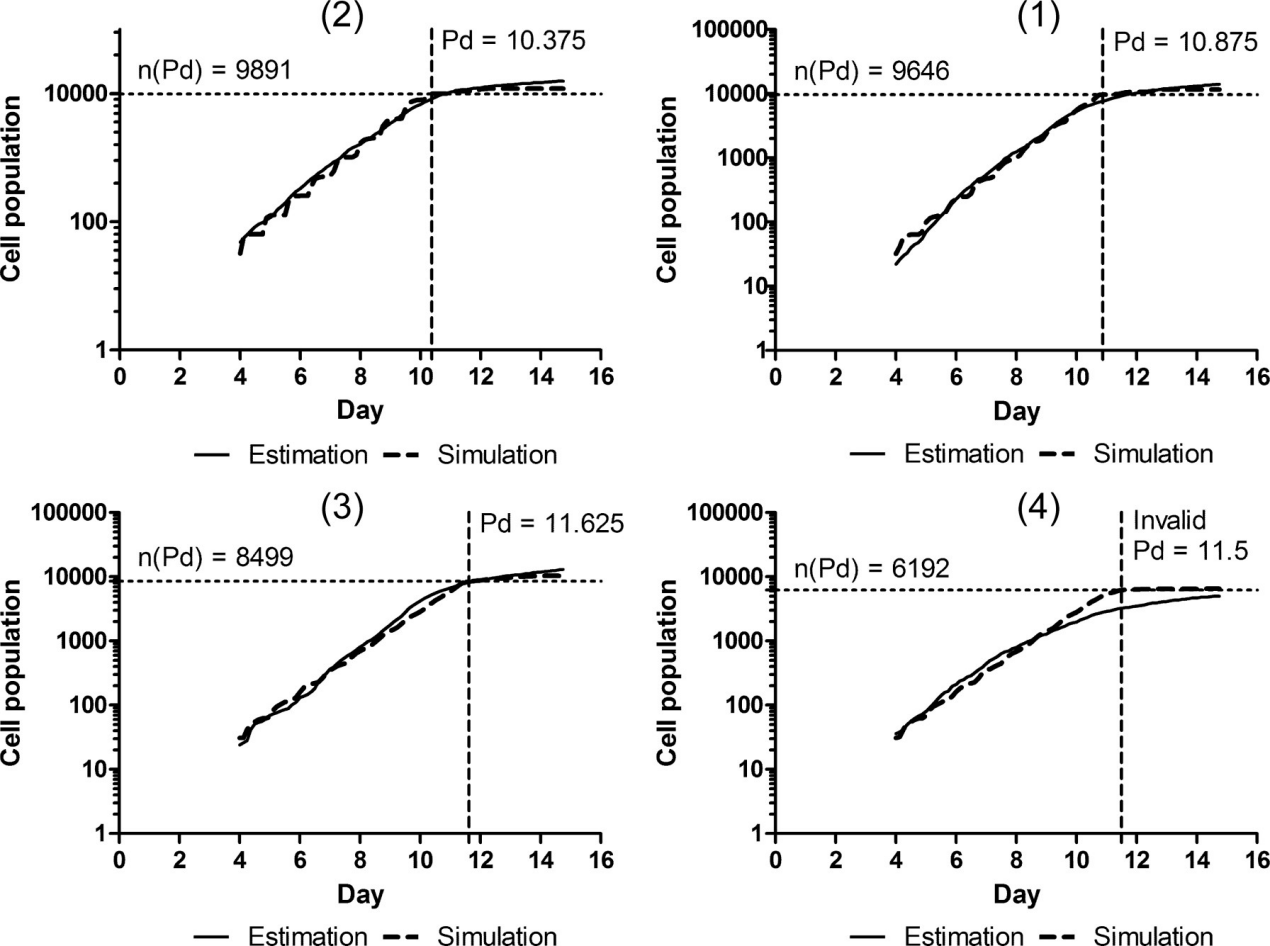
Growth curves in estimation and simulation. The bridge adjusted the simulated growth curves approximately to the estimated ones through the optimization of relaxation coefficients for points (1)–(4). The day to reach the plateau phase was calculated as 10.875, 10.375, 11.625, and 11.5; the number of cellular particles on the day was 9646, 9891, 8499, and 6192; and the error function value was 0.094, 0.055, 0.055, and 0.101 for points (1)–(4), respectively. For (4), the day was invalid because the adjustment was insufficient.

Fig 5 shows the growth curves obtained from estimation and simulation for the representative points. The bridge enabled the relaxation coefficients to adjust the simulated growth curves to the estimated ones for spheroids (1)–(3). The adjustment appeared insufficient to fit two growth curves of spheroid (4) during the transitional phase. However, the final number of cells was almost the same in both growth curves. In addition, Fig 5 shows that the sooner the growth curve reaches the plateau phase, the more quickly the cell population grows. For the feature plane, the cell population grows the quickest in descending order of the second, first, third, and fourth quadrants.

### Verification of spheroids *in silico*

**Fig 6:**
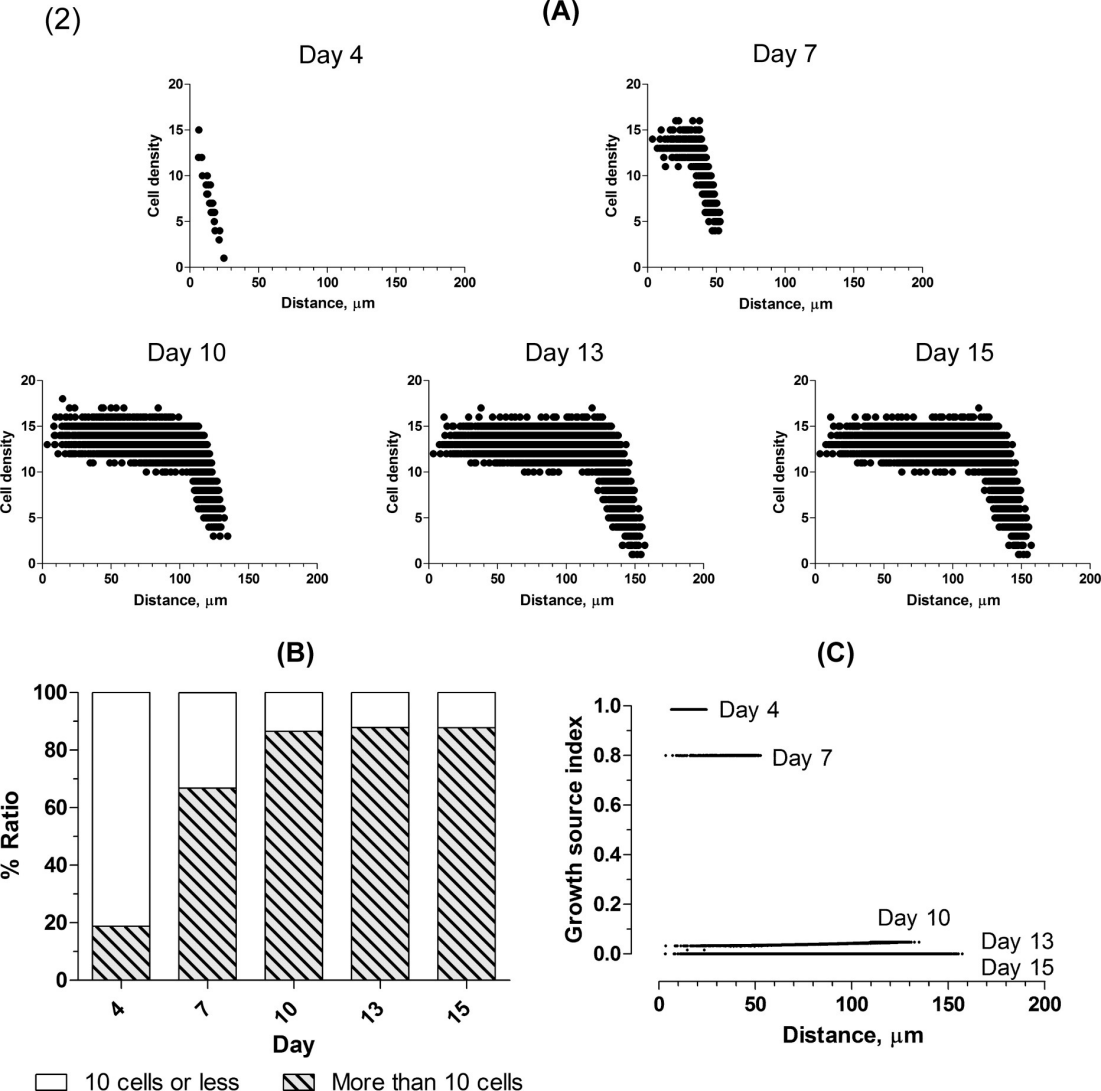
Cell density profile in the spheroid *in silico* (2). **(A)** The horizontal axis shows the distance from the center and the symbol • denotes a region where the attractive force acts. The vertical axis shows cell density in the regions. The cell density gradually reaches the kissing number 12 at the center on day 13. Outside the center, numerous regions exist with cell density more than 12 because the density was increased by 1 even when the region partly included the cellular particle. Finally, the cell density decreases rapidly toward the surface. Over time, the range of cell density narrows around the center. **(B)** The ratio of more than 10 cells increases rapidly up to day 10 and is subsequently maintained. **(C)** Growth source index for each region is plotted on the same horizontal axis as in **(A)**.

Each spheroid *in silico* on day 15 consisted of cellular particles bounded by a simple cubic structure (lower) and a hexagonal close-packed structure (upper) (Table 2). As an example of spheroids *in silico*, Fig 6A illustrates the cell density profiles of the spheroid *in silico* (2) in Fig 5. The diameter is given in Table 2. The cell density can be seen to have maintained a mostly hexagonal close-packed structure. The cell density was approximately 12 and decreased toward the surface. In addition, as Fig 6B shows, the period until day 10, in which the ratio of more than 10 cells increases rapidly, corresponds to the exponential phase in Fig 5 (2). Fig 6C illustrates that the growth source index decreases to keep pace with increases in the ratio and then is exhausted on day 13, so that the ratio reaches a plateau with respect to the increase. In addition, the index increases slightly toward the exterior for the spheroid *in silico* until day 10.

**Table 2:** Spheroids *in silico* on day 15.

	(1)	(2)	(3)	(4)
Number of cellular particles	11594	11990	10357	6506
Lower bound	9312	9569	7718	5140
Upper bound	13172	13536	10917	7271
Diameter [μm]*	289	291	271	237

*Scale *in silico*

Moreover, as shown in Table 3, the spatiotemporal relative relationship with regard to the diameters of spheroids was generally maintained between spheroids *in vitro* and *in silico*. The descending order of their size *in silico* [(2), (1), (3), and (4); shown in Table 3A] is consistent with that of the quickness of cell population growth shown in Fig 5.

**Table 3:** Spatiotemporal relative relationship between spheroids *in vitro* and *in silico*.

(A)	(1)	(2)	(3)	(4)
*In vitro*	0.93	1	0.94	0.74
*In silico*	0.99	1	0.93	0.81
**(B)**	(1)	(2)	(3)	(4)
*In vitro*	28.64	30.66	28.92	22.70
*In silico*	28.87	29.14	27.12	23.68

For the diameter on day 15, **(A)** demonstrates an index of the relative spatial relationship that is the ratio of each spheroid (1)–(4) to spheroid (2), and **(B)** demonstrates an index of the relative temporal relationship that constitutes the growth ratio from the first day for each spheroid.

### Distribution of the generation in spheroids *in silico*

**Fig 7:**
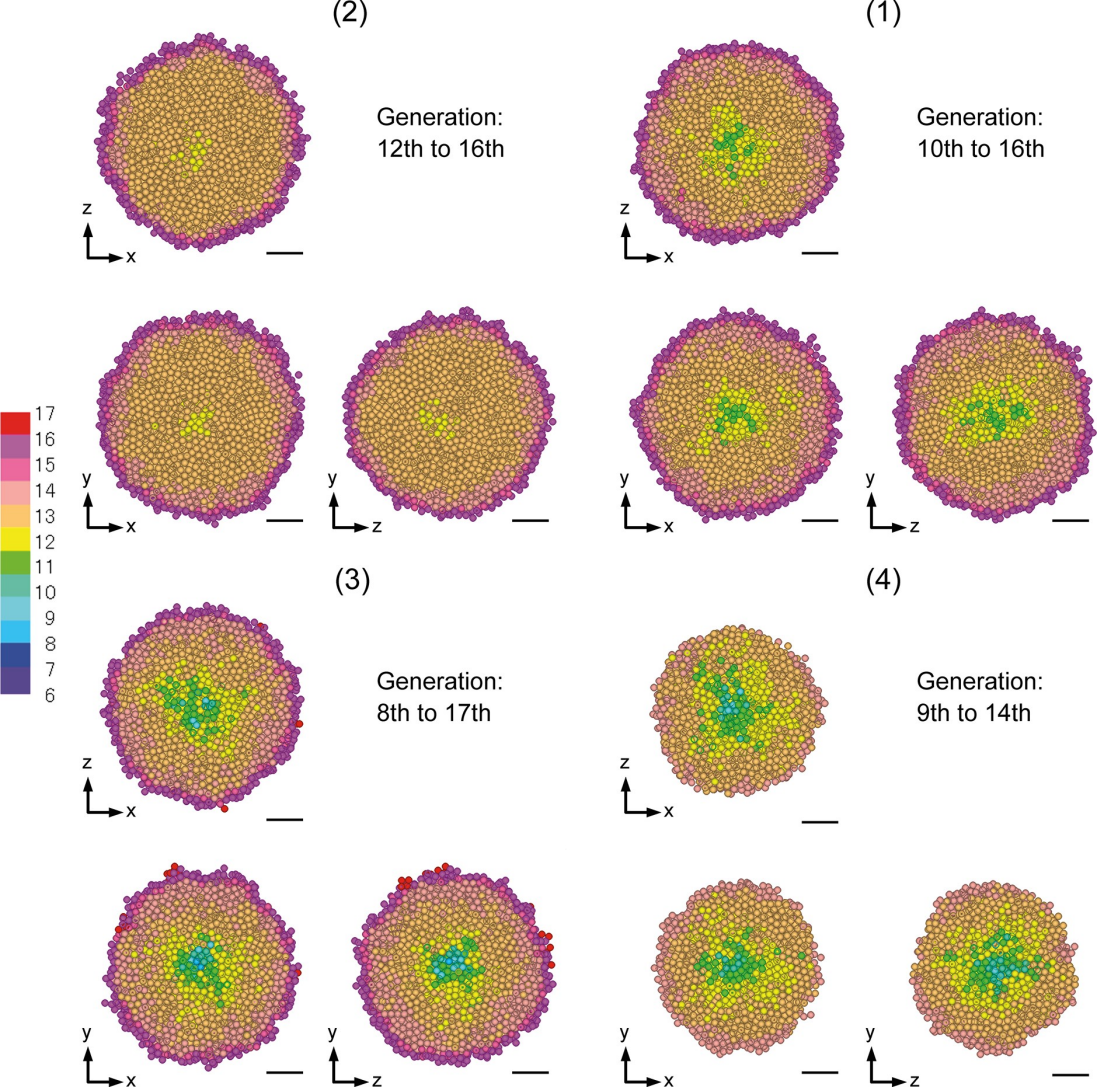
Generation profiles of spheroids *in silico* on day 15. The spheroids are presented by three orthogonal cross sections through their center at the position corresponding to their own quadrant. The bars represent 50 μm *in silico*.

Fig 7 shows three orthogonal cross sections through the center of spheroids *in silico* (1)–(4), which comprise generation profiles colored based on the generations assigned to cellular particles. The descending order of their section sizes [(2), (1), (3), and (4)] is consistent with that of their size *in silico* given in Table 3A. Both the range and distribution of the generations are different based on the quadrant. As expected, each spheroid has its most recent generations near its surface, consistent with the knowledge that the frequency of cell division increases toward the surface [19]. The generation number represents the degree of proliferative activity, which is high for the proliferation zone and low for the quiescent zone including the central necrotic core. The complexity of various generations around the center appears more clearly in the descending order of spheroids *in silico* (4), (3), (1), and (2), which is equivalent to that of the slowness of cell population growth as shown in Fig 5. By contrast, the generation mixture appears more simply in the descending order of spheroids *in silico* (2), (1), (3), and (4). In addition, generations of spheroid (4) were assigned as being younger and more uniform, because the simulated growth rate was more rapid than the estimated growth rate (see (4) in Fig 5). The relationship between the growth rate and the generation mixture is described later in detail.

**Fig 8:**
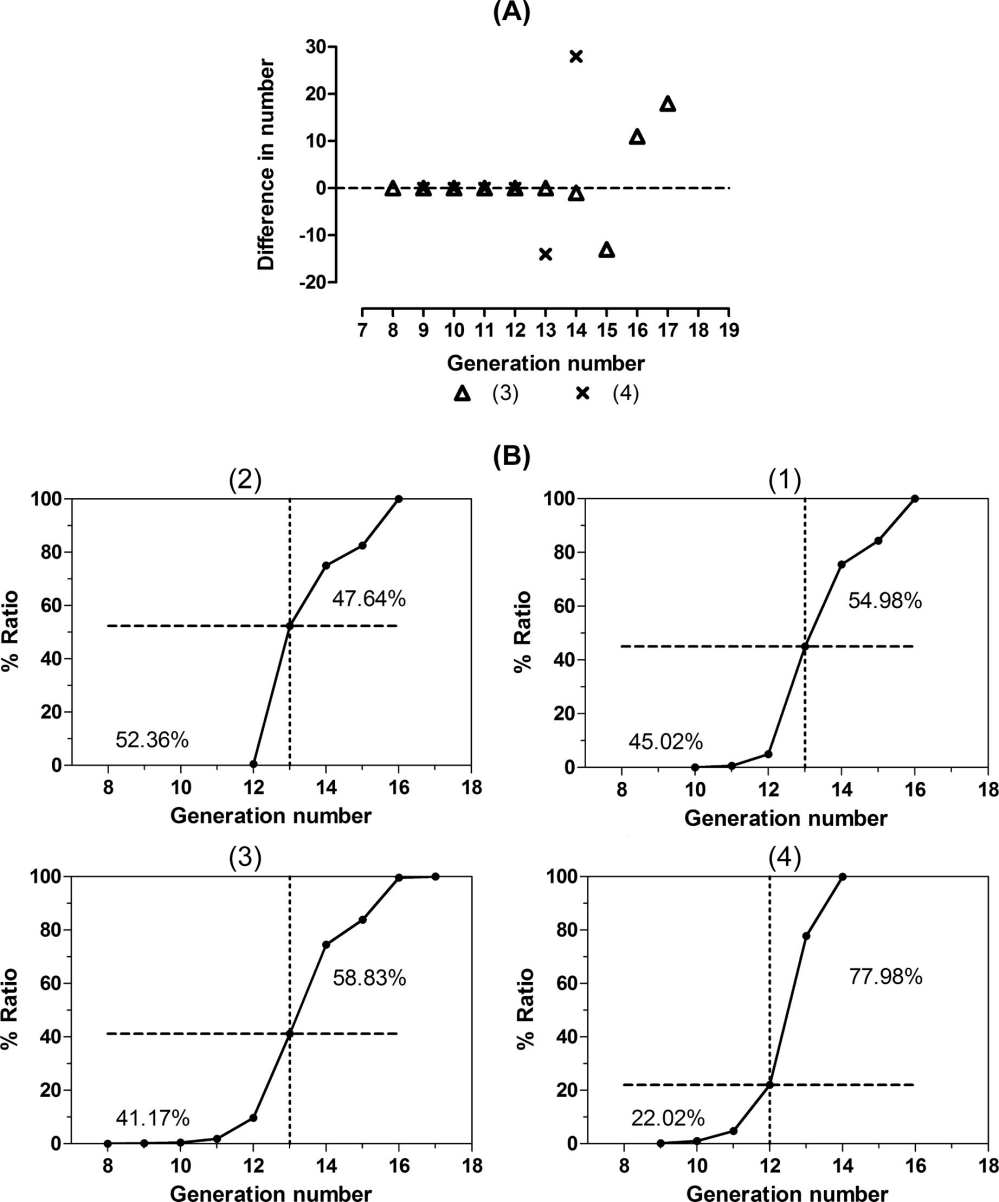
Cumulative ratio of the generation in spheroids *in silico* on day 14. **(A)** Difference in the number of cellular particles between day 14 and day 15 plots for each generation. **(B)** The dashed line represents the cumulative ratio of generations with low activity and the dotted line illustrates the upper limit. The ratio of generations with low virtual inner proliferative activity is shown under the dashed line and that of generations with high virtual inner proliferative activity is shown over the line.

Fig 8A shows that for the spheroid *in silico* (3), cellular particles of the 14th and 15th generation are decreased and those of the 16th and 17th generations are increased. Virtual inner proliferative activity was defined as low when no difference occurred in the number of cellular particles in that generation and as high when there was a difference. Up to the 13th generation, the virtual inner proliferative activity should be low; therefore, the ratio is 41.17% as shown in Fig 8B (3). Comparing spheroids *in silico* (1) and (2) on day 13, no difference was detected in the number of cellular particles. These cells in the spheroid *in silico* (2) were unable to divide, because growth sources were exhausted on day 13 as shown in Fig 6C. This was also the case for spheroid *in silico* (1), because the growth curves in Fig 5 exhibit similar transitional phases between spheroids *in silico* (1) and (2). In addition, growth curves were calculated to reach the plateau phase on days 10.875 and 10.375 for spheroids *in silico* (1) and (2), respectively. These are slightly earlier than day 11.625 for spheroid *in silico* (3). Their generation ratios with low activity must be greater than those for spheroid *in silico* (3) because the growth source index decreases earlier. If the virtual inner proliferative activity is low up to the 13th generation, the ratios are 45.02% and 52.36% for *in silico* spheroids (1) and (2), respectively, which are slightly larger than the ratio of 41.17% for *in silico* spheroid (3). If the activity is low up to the 14th generation, both ratios are over 70%. Given this, the growth curves must reach the plateau phase much earlier than they do for *in silico* spheroid (3), which is inconsistent with the growth curves. Therefore, the activity should be low until the 13th generation. For *in silico* spheroid (4), Fig 8A shows that the number of cellular particles decreases in the 13th generation and increases in the 14th generation. Until the 12th generation, the activity should be low. Thus, spheroids *in silico* were given their virtual inner proliferative activity according to the generation, which was assigned to each cellular particle.

Fig 8B also shows the rate of cell population growth depending on the generation range. A higher rate of growth would lead to a graph of near-linear cumulative generation ratio over a narrow range of generations. As the rate of growth slows, the graph would transform gradually to a sigmoid-like curve over a wider range of generations. As the growth rate reduces, older generations remain and the ratio of generations with high virtual inner proliferative activity (“high”) is greater.

For the feature plane, the descending order of complexity of the generation mixture [(4), (3), (1), and (2)] is equivalent to that of ratio of the high, which corresponds to that of slowness of the growth rate. In other words, the descending order of quickness of the growth rate [(2), (1), (3), and (4)] is also equivalent to that of ratio of the generations with low virtual inner proliferative activity, which corresponds to that of simplicity of the generation mixture.

In addition, the ratio of the high shown in Fig 8B is roughly consistent with 0.60, the ratio of cell volume labeled with [^3^H]thymidine to total volume, which Folkman *et al*. demonstrated using a 1.0 mm diameter and 20-day-old V-79 spheroid grown in soft agar [30]. For several tumor cell lines, spheroid diameters were shown to be inversely proportional to the number of spheroids in a flask, and isolated spheroids reached a maximum mean diameter beyond which no further expansion was possible for self-regulation of the cell population in 3D culture.

### Virtual inner proliferative profiles for spheroids *in silico*

**Fig 9:**
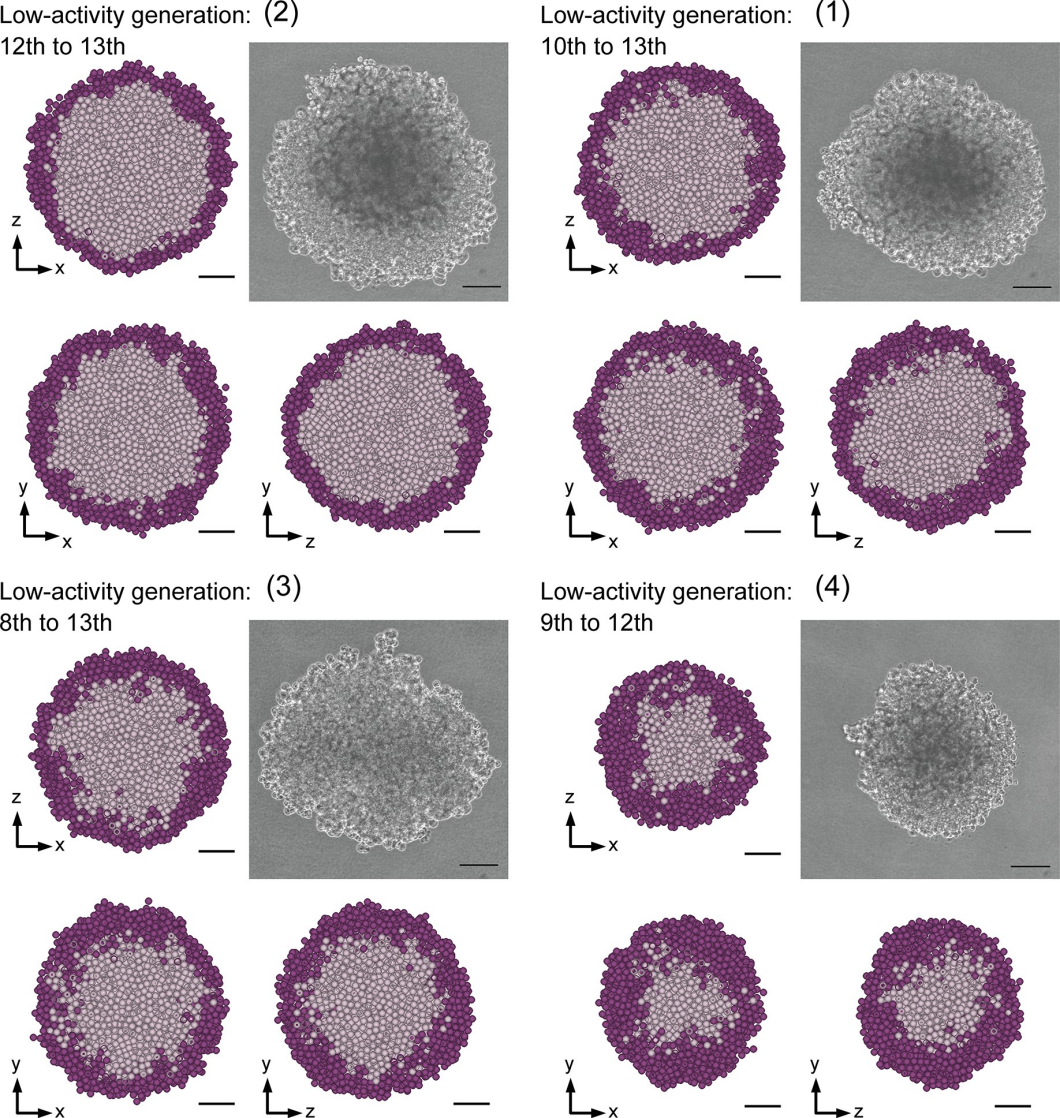
Spheroids *in vitro* on day 14 and their virtual inner proliferative profiles. Spheroids *in vitro* (1)–(4) are presented with their provided virtual proliferative profiles at three orthogonal cross sections through the center of the spheroids *in silico* computed based on their microscopy time-lapse images, where cellular particles are depicted with a light color for the low and dark for the high. The bars represent 100 and 50 μm for spheroids *in vitro* and *in silico*, respectively.

Fig 9 demonstrates spheroids *in vitro* and their corresponding virtual proliferative profiles, given by their virtual inner proliferative activity. The virtual proliferative profiles were consistent with spheroid schematics, which involved a typical zone of cell proliferation, as reported in a previous study [19]. Furthermore, the relative relationship with regard to the size was generally maintained between spheroids *in vitro* and *in silico* in Table 3A. Fig 9 shows that the smaller the size becomes, the thicker the layer with high virtual inner proliferative activity also becomes. In other words, the slower the cell population grows, the smaller the ratio of low virtual inner proliferative activity becomes.

**Fig 10:**
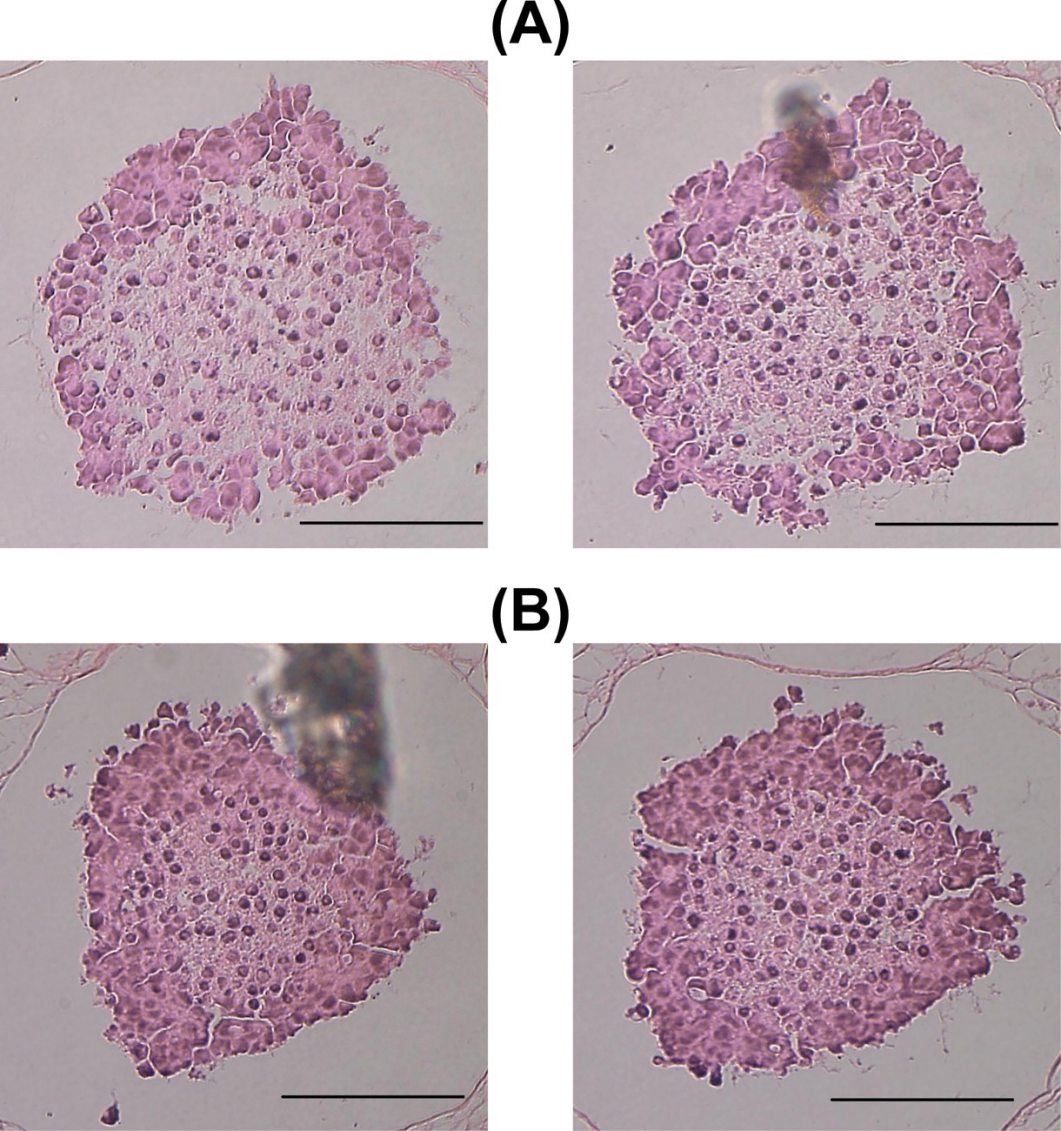
Histochemical profiles of HeLa spheroids on day 14. Spheroids were chosen to be as round as possible though their cross sections for every 20 μm. The sections stained with hematoxylin and eosin are shown as a consecutive pair including the largest size, and have a dark exterior and a light interior on the ground color. Scale bars are 100 μm. In cross sections associated with the largest size, both regions of the spheroid and the light color interior were approximated into two different circles covering each contour. Using the volume of spheres derived from the circles, the ratios of the quiescent zone to the whole area were approximately estimated as **(A)** 51.2% and **(B)** 26.2%. In **(A)** and **(B)**, the proliferation zone ratios are inversely 48.8% and 73.8%, respectively.

Fig 10 shows the histochemical profiles around the center of HeLa spheroids incubated for 14 days with a seeding density of 5000 cells/well. The density was increased to select clearer cross sections from many spheroids. The spheroids are slightly smaller than those in Fig 9 because the seeding density was higher. This observation is in accordance with the study of Folkman *et al*. [30]; i.e., the size of spheroids was inversely proportional to the number of spheroids in a flask. In addition, regarding the size, a combination of cell-cell interactions and cell–matrix interactions has been shown to contribute to the compaction of spheroids in previous work [31–34].

The ratio and distribution of high virtual inner proliferative activity in spheroids *in silico* (1)–(3) in Fig 8B and Fig 9 are similar to the proliferation zone ratio and histochemical profiles shown in Fig 10A. This also holds true for spheroid *in silico* (4) in Fig 8B, Fig 9 and Fig 10B. These results demonstrate that the virtual inner proliferative activity given to spheroids *in silico* on day 14 is compatible with the histochemical profiles of cross sections around the center of spheroids *in vitro* on the same day. Moreover, the histochemical profiles shown in Fig 10A were similar to those shown in the V-79 spheroids, and the proliferative zone ratio of 48.8% estimated from the spheroids in Fig 10A was close to the proliferative volume ratio of 0.60 of V-79 spheroids. Therefore, the ratio of the high virtual inner proliferative activity given by the generation of the spheroids *in silico* (1)–(3) is consistent with the proliferative volume ratio of V-79 spheroids.

## Discussion and conclusion

We have developed the first step toward an *in silico* analysis method to bridge the gap between experiments and simulation. HeLa spheroids were analyzed through spheroids *in silico* optimally replicated by 3D growth simulation created for soft agar culture. The present method involved generating a growth curve from time-lapse images of spheroid growth, growth simulation with several mathematical models, and a bridge between spheroids *in vitro* and those *in silico*. The latter allowed us to characterize spheroids *in vitro* on the feature plane with the growth curves and replicate those growth curves *in silico* by optimizing parameter values in the model. The bridge enabled the feature plane to be applied to spheroids *in silico*. Moreover, analyzing the generations assigned to cellular particles allowed us to assign virtual inner proliferative activity to spheroids *in silico*. This method was validated and found to be consistent with the histochemical profiles of HeLa spheroids grown under the same conditions.

As for cross-fertilization of real cell imaging and virtual cell imaging through modeling, Ulman *et al*. described that virtual cell imaging provided the ability to improve the quality of the algorithms applied to real cell image analysis [35]. By contrast, a distinguishing feature of the *in silico* analysis method is to promote understanding of actual spheroids by means of those *in silico*, embodied by growth simulation based on time-lapse images from the researcher’s own samples.

In the present work focusing on individual HeLa spheroid growth in soft agar culture, experimentally observed spheroid growth curves could be well characterized by two principal components and classified into four types according to the quadrant of the feature plane. Although growth simulation successfully regenerated the growth curves plotted on the first, second, and third quadrants, that on the fourth was slightly out of alignment, especially in the transitional phase (see (4) in Fig 5). Preliminary trials suggested that the cell cycle model improved replicas in the transition phase depending on the quadrant. Therefore, further refinement of the model would achieve more precise simulation of spheroid growth; this will constitute an important subject of our future work.

The generation profiles in spheroids *in silico* showed differences depending on the quadrant to which they belonged (see (1)–(4) in Fig 7). The complexity of the generation mixture is formulated as (2) < (1) < (3) < (4), which notably coincides with the converse of growth rates with a formulation of (2) > (1) > (3) > (4) (see Fig 8B).

The relationship of the quadrant in terms of complexity agrees with both the descending order of high virtual inner proliferative activity [(4), (3), (1), and (2)], and the ascending order of their diameter [(4), (3), (1), and (2)] (see Fig 8B and Table 2). These observations suggest that a spheroid that proliferates quickly has a larger surface, so that growth sources are simultaneously supplied to more cells, cell division is more synchronized, growth sources are exhausted more rapidly, and the number of cells with low virtual inner proliferative activity increases.

The ratio and distribution of virtual inner proliferative activity in Fig 8B and Fig 9 are similar to the proliferation zone ratios and histochemical profiles in Fig 10. Note that these are similar to those demonstrated using V-79 spheroids [30].

The results obtained from the analysis of individual spheroid growth suggest that the inner proliferative activity of HeLa spheroids can be predicted through 3D simulations *in silico* based on time-lapse imaging of HeLa cells in soft agar culture. This also suggests that time-lapse images of spheroid growth can provide accurate virtual inner proliferative activity for spheroids *in vitro*; thus, the relationship *in silico* between the virtual inner proliferative activity and the rate of spheroid growth allows us to reveal the relationship *in vitro*. This strongly supports the fact that our framework, assumptions, and modeling are appropriate for the investigation of living spheroids. In particular, soft agar culture contributes greatly to the performance of the first step toward the *in silico* analysis method, because spheroids are formed only by proliferation.

Different culture techniques based on the concept of biofabrication have been developed for the formation of spheroids, such as the hanging drop method, rotating wall container, and micro-mold technology [36]. The *in silico* analysis method can be performed on these cultures if bases have been formed, including the acquisition of time-lapse images of the formation process, tracking of a spheroid through the images, and mathematical modeling of the formation process caused by both proliferation and mechanisms specific to the culture techniques. Building the bases is a prerequisite of the *in silico* analysis method.

Therefore, even if it is limited to soft agar culture at the moment, the *in silico* analysis method will facilitate the expansion of the application scope of this method for the first step. Potential modifications for the control include the analysis period being extended after medium exchange when the growth source is exhausted, and cell culture changed to plain media without growth supplements. In addition, expanding this to experiments with a drug such as anticancer drugs to inhibit cell growth would require analysis of the collapse of the dose-response relationship on spheroids. Sensitivity regarding both the appearance and the virtual inner proliferative activity of spheroids will allow measurement along the time course from initial drug addition. The sensitive measures are complementary to conventional agent measurement following drug testing, allowing for more detailed information to be extracted from the test. Moreover, spheroids will evolve into co-cultured spheroids and even organoids. If the information extracted from such tests and the knowledge of cellular receptors, proteins, and gene expression can be integrated to create a response model for drug delivery and efficacy, such models will be introduced to the *in silico* analysis method to permit more extensive and refined prediction.

Hence, we believe that the concept of *in silico* spheroid analysis can be effectively used for cell biology experiments on tissue cell cultures. Such analyses with virtual embodiment computed using a researcher’s own samples will reduce the gaps between real experiments and simulations. Therefore, additional research is urgently required to analyze cell populations, extend the range of cell lines, improve mathematical models, and increase computing power. A long-term challenge is the extension to culture systems for biofabrication. In turn, the *in silico* analysis method might facilitate drug discovery and the assessment sensitivity for drug delivery and efficacy testing for various diseases including cancers.

## Supporting information

S1 Table Soft agar assay protocol. (DOCX)
