# Supplementary material for: Time-lapse imaging of HeLa spheroids in soft agar culture provides virtual inner proliferative activity
Source: PLoS One. 2020 Apr 17;15(4):e0231774. doi: 10.1371/journal.pone.0231774 (PMC7164667; doi:10.1371/journal.pone.0231774)
Supplement: S1 Table — (DOCX) [file pone.0231774.s001.docx]

**S1 Table. Soft agar assay protocol.**

| **Step** | **Parameter** | **Value** | **Description** |
| --- | --- | --- | --- |
| 1 | Lower agarose | 0.5 mL | 0.7% agarose |
| 2 | Cells in upper agarose | 5.0 mL | 0.35% agarose |
| 3 | Incubation | 37 °C 24 h |  |
| 4 | Feeding | 5 mL | Growth medium |
| 5 | Assay readout |  |  |
| **Step Details** |  | | |
| 1 | Sterile double concentration RPMI 1640 growth medium (32.4 g/L RPMI 1640 medium powder, 4.0 g/L NaHCO_3_, 4 mM GlutaMAX-1, 200 units/mL penicillin, 200 μg/mL streptomycin, and 20% FBS) was warmed at 42 °C in a water bath. Next, 1.4% agarose solution was autoclaved and then precooled at 42 °C. Equal volumes of growth medium and agarose solution were mixed to obtain a 0.7% agarose medium and stored 42 °C. Agarose medium (0.7%) was spread into 6-well plates and solidified. | | |
| 2 | Cells (200 cells/mL) were pre-warmed in RPMI 1640 growth medium (2.5 mL, 100 unit/mL penicillin, 100 μg/mL streptomycin, and 10% FBS) at 42 °C. Cells were added to the 0.7% agarose medium and seeded on the agarose medium layer in the plate. | | |
| 3 | 0.35% agarose medium containing the cells was solidified at 37 °C for 24 h. | | |
| 4 | Cells were fed with growth medium (5 mL) and cultured at 37 °C in 5% CO_2_ (BioStation CT). | | |
| 5 | Images of the cells were captured at intervals of 3 h for 14 days. | | |
